# Supplementary material for: Mechanically-tunable bandgap closing in 2D graphene phononic crystals
Source: NPJ 2D Mater Appl. 2023 Feb 23;7(1):10. doi: 10.1038/s41699-023-00374-4 (PMC11466820; doi:10.1038/s41699-023-00374-4)
Supplement: Supplementary file 1 — Supplementary Information [file 41699_2023_374_MOESM1_ESM.pdf]

Supplementary Information: Mechanically-tunable bandgap closing in 2D graphene phononic crystals

Jan N. Kirchhof[1] and Kirill I. Bolotin[1]**

[1] Department of Physics, Freie Universität Berlin, Arnimallee 14, 14195 Berlin, Germany

*jan.kirchhof@fu-berlin.de *kirill.bolotin@fu-berlin.de

Supplementary Note 1: Band structure calculations for an infinite lattice

The infinite model for the phononic band structure calculations is based on two studies within the same FEM simulation model (Comsol Multiphysics 5.5). In the first step we simulate the tension redistribution upon pattering (stationary study). We then use the calculated tension distribution as input for a second study step (eigenfrequency study). In this step, we parameterize the x and y component of the k vector to cover the 1.BZ, and implement them into the model as periodic boundary conditions (Floquet) along the outline of the unit cell. We then calculate and plot the eigenfrequencies, what gives us the band structure. For more details see Ref. [1]. To assure that our simulations properly capture the phononic band gaps, we extend our calculations to more high symmetry points. For the unit cell shown in Supplementary Figure 1a, we plot the extended band structure for $\sigma_{\mathrm{xx}}/\sigma_{\mathrm{yy}} = 1$ and $\sigma_{\mathrm{xx}}/\sigma_{\mathrm{yy}} = 1.7$ inside the 1.Brillouin zone, see Supplementary Figure 1b,c. We find that the valence band maximum between Γ and X and the conduction band minimum at X, and it is therefore sufficient to focus on the high symmetry points shown in the main paper.

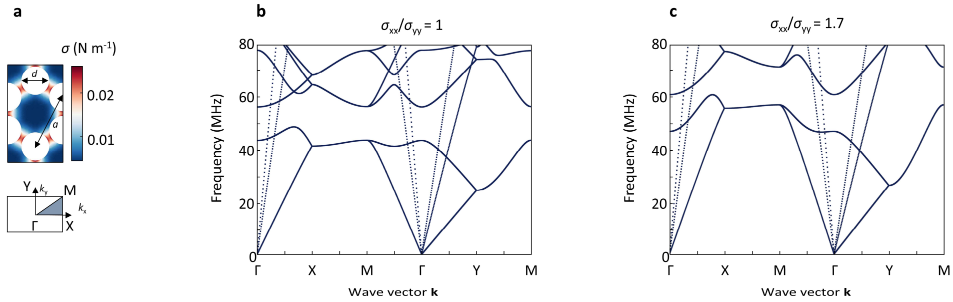

Supplementary Figure 1 | Extended band structure. a, Unit cell of the honeycomb lattice with redistributed tension (top) and the corresponding first Brillouin zone (bottom). **b,c**, Extended phononic band structure for the unit cell shown in (a) with entirely uniform tension (σ_{xx}/σ_{yy} =1.) and implemented uniaxial tension σ_{xx}/σ_{yy} =1.7.

Supplementary Note 2: Transmission studies

To perform our transmission studies, we use a pre-stressed frequency domain study. In this study the phononic device is clamped along its perimeter (Supplementary Figure 2) and in a first study step we again calculate the tension redistribution upon patterning. In a second step, we add a time depended pressure in z direction at area A, which simulates an optothermal drive. For the transmission study, we then sweep the frequency of this time depended perturbation and calculate the response of the entire geometry (compare Eq. 1 main text). For this study step, we add isotropic damping ($\eta = 0.01$) to the graphene, which reproduces the quality factors ($Q \sim 100$) typically observed for graphene resonators at room temperatures. To simulate the effect of electrostatic pressure to the transmission studies, we add a boundary load to the entire device (including A and B) in z-direction and repeat the frequency sweep.

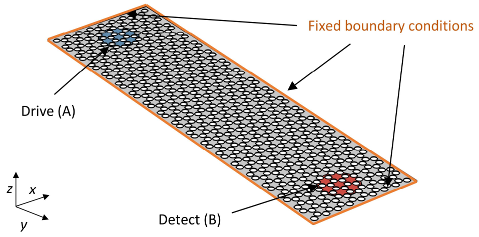

Supplementary Figure 2 | Transmission geometry. The phononic device is clamped at its perimeter via fixed boundary conditions and motion is excited at A (blue) and detected at B (red).

Supplementary Note 3: Transmission circular reference device

As a comparison to the presented bandgap closing in the main text we perform transmission studies on a circular device under applied pressure which is excited at its centre and probed on the outside. The device geometry and resulting transmission spectra are shown in Supplementary Figure 3a,b, where we find clear bandgap features (shaded area) with (red) and without (blue) applied pressure. In circular devices we find band gap features up to at least 30 kPa applied pressure in agreement with previous work.[1]

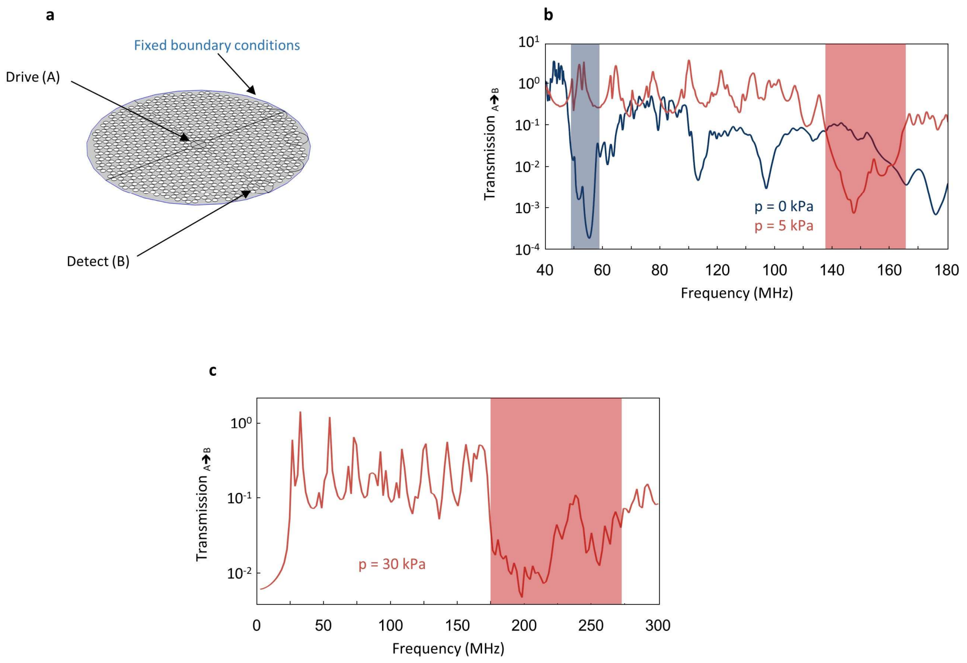

Supplementary Figure 3 | Transmission circular reference device. a, Transmission geometry for a rectangular phononic device. At point A mechanical motion is excited by a frequency modulated laser, which than travels through the device and is detected at point B by a second laser spot. **b,c** Transmission from A to B vs. excitation frequency for the device shown in (a) for 0, 5 and 30 kPa applied pressure. A clear bandgap region is visible for all cases.

Supplementary Note 4: Phononic bandgap vs. uniform residues

In addition the added pieces of mass (Figure 4 main text), we also investigate the effect of a uniform layer of resist, which could be present on a device after thermal annealing. In Supplementary Figure 4 we plot transmission for a device made from clean graphene (blue) and one contaminated with a 3 nm layer of PDMS (green). For both cases we find a clear bandgap, the added PDMS however causes a downshift in frequency as the entire device becomes heavier. Also the bandgap is slightly less pronounced, but still clearly noticeable. For PDMS we assume a Young's modulus of 0.75 MPa, a Poisson's ratio of 0.49 and a density of 970 kg m^{-3}.

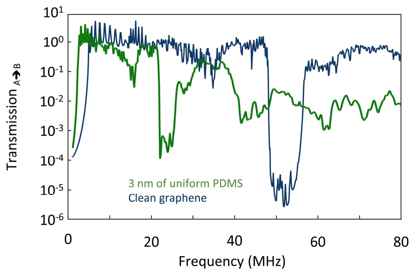

Supplementary Figure 4 | Phononic bandgap vs. uniform residues. Transmission vs. frequency for a phononic device made from clean graphene (blue) and with a 3 nm uniform layer of PDMS residues (green).

Supplementary Note 5: Phononic bandgap vs. tension disorder

To represent a random but smooth enough spatial tension distribution in our devices we use a sum of plane waves with randomized amplitude $a(m,n)$ (between -1 and 1) and phase $\phi(m,n)$ (between 0 and π) of each mode:

$$\sigma(x,y) = p\sum_{\mathrm{m}=-\mathrm{M}}^{\mathrm{M}}\sum_{\mathrm{n}=-\mathrm{N}}^{\mathrm{N}} a(m,n)\cos(2\pi(mx+ny)+\phi(m,n)) \quad (1)$$

The factor p controls the disorder strength.

Supplementary Note 6: Phononic bandgap vs. hole size variations

We also investigate the effect of disorder within the phononic pattern in terms of a) variations of the radius of individual holes (so that the holes are not perfectly round) or b) size variations between the different perfectly circular holes that make up the phononic crystal.

Before we simulate the effect of a) and b) on the phononic bandgap, we need to get a feeling for the experimentally relevant disorder in patterned phononic crystals. To this end, we look at a real phononic crystal made from ~5 layer thick graphene (Supplementary Figure 5). We patterned this sample using the honeycomb lattice described in the manuscript by focused ion beam milling (FIB). The spatial resolution of this process is in the order of ~20 nm, which is compared to the hole diameter (500 nm) rather small, and we expect high precision and reproducible hole diameters. And indeed, we find a regular patterned phononic crystal as shown in Supplementary Figure 5.

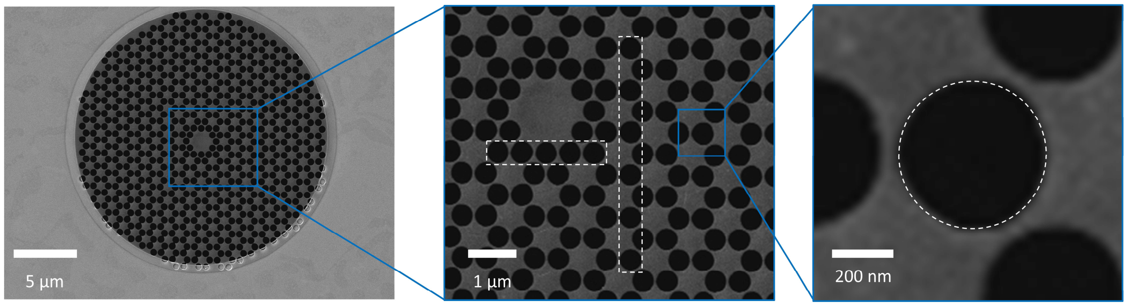

Supplementary Figure 5 | Circular prototype device made from 5 Layer graphene for experimental work. The phononic pattern is regular and the variations in hole size are small.

Next, we study the effect of non-circular holes (a) in our infinite model. We start by replacing two holes in the unit cell by ellipses (90° rotated to each other, eccentricity: $e = \sqrt{1 - \left(\frac{d_1}{d_2}\right)^2} = 0.574$, as shown in Supplementary Figure 6c) and calculate the corresponding band structure (Supplementary Figure 6d). If we compare this to the reference band structure obtained with perfect holes (Supplementary Figure 6a,b), we find that some bands split into closely lying sub-bands. Nevertheless, the bandgap remains. No change in size or position of the bandgap is noticeable. To go a step further, we try to capture variations in hole radius of individual holes comparable to the sample

shown above (Supplementary Figure 5 right). To do so we implement exaggerated "wobbly" holes into the unit cell (Supplementary Figure 6e). Again, we find no major impact on the bandgap region (Supplementary Figure 6e) and thus the robustness of our results.

To check the effect of variations in hole size between different holes (b), we model a structure were the size of randomly picked holes ("defects") is decreased or increased by 10%. In Supplementary Figure 6g, we show the used structure with a defect density ($\frac{N_{\text{defect}}}{N_{\text{holes}}}$) of 12.5%. If we zoom in on a device with larger defect density of $\frac{N_{\text{defect}}}{N_{\text{holes}}} = 50\%$, the variations in hole size are clearly visible (see Supplementary Figure 6h). Next, we simulate the transmission for devices with defect densities from 0 to 50% (Supplementary Figure 6i). We find that the bandgap clearly persists to a defect density of at least 50%. This level of disorder is clearly much higher than that in experimentally achieved devices (Supplementary Figure 5). We therefore conclude that we can fabricate the devices ordered enough to observe the phenomena we study in the main text.

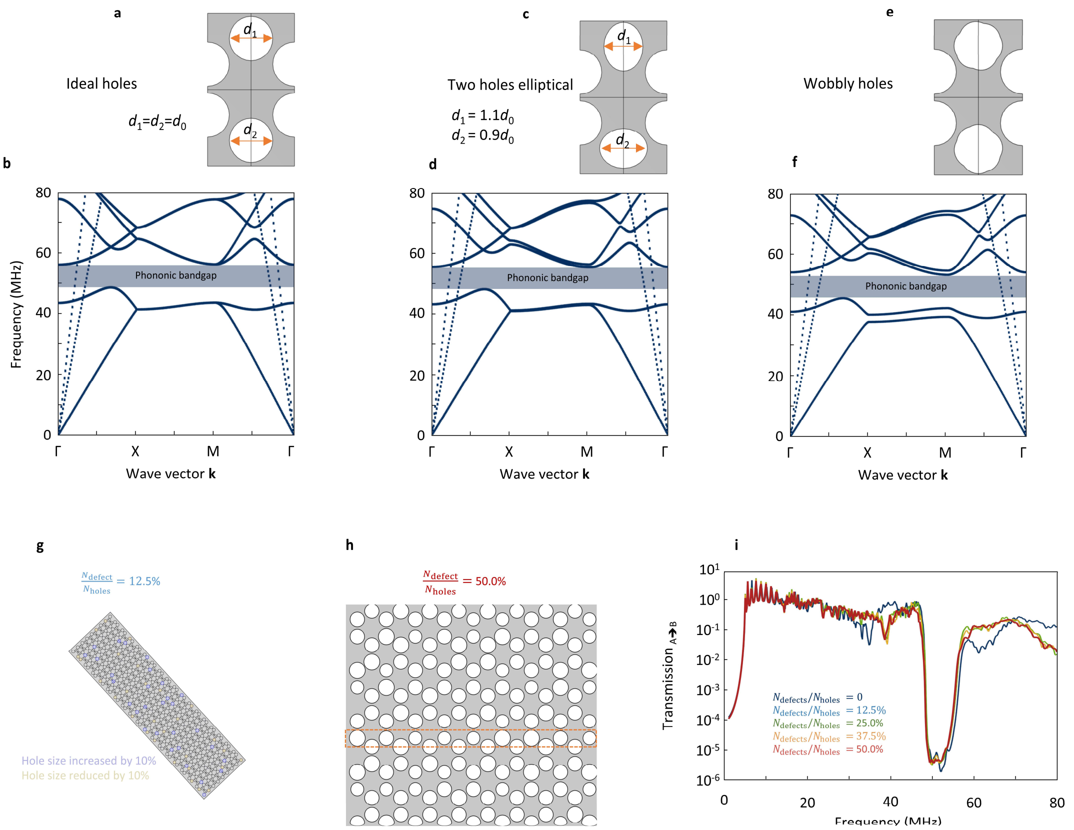

Supplementary Figure 6 | Phononic bandgap vs. hole size variations a,b Unit cell and corresponding band structure calculation for ideal holes (same as in main text Supplementary Figure 1b) **c,d** Unit cell with two elliptical holes and corresponding band structure calculation. The phononic bandgap remains almost unchanged. **e,f** Unit cell with two wobbled holes and corresponding band structure calculation The phononic bandgap remains almost unchanged. **g,h** Finite model with different degree of lattice defects (hole diameter variations) implemented. The blue (yellow) marking indicates holes, which will be increased (decreased) in size by 10%. **i** Transmission simulation for varying defect density. For a defect density of 50% the bandgap is clearly distinguishable in the transmission plots.

Supplementary Note 7: Phononic band gap vs. layer number

As a potential approach to overcome challenges associated with the fabrication of uniform and residue free suspended graphene devices for phononic pattering, we suggest to use thin multilayers. For this we have to check, if the bandgap persist also for thicker devices. We thus perform band structure calculations for various thickness (maintaining a constant stress in the device) and extract the band gap (Supplementary Figure 7). We find a bandgap up to ~350 layers of graphene.

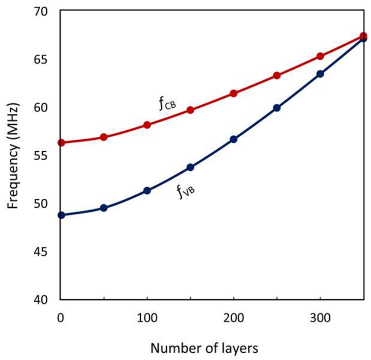

Supplementary Figure 7 | Phononic band gap vs. layer number. Valence band maximum (f_{VB}) and conduction band minimum (f_{CB}) vs. number of graphene layers extracted from band structure calculations.

Supplementary Note 8: Localization-delocalization transition

To further highlight the usefulness of the proposed system and to strengthen the analogy to a MIT, we perform an additional study. In it, we examine the localization of defect vibrational modes that can be compared to the localization of mid-gap defect states in semiconductors. By varying the size of the bandgap using our tension engineering approach, we observed the behaviour similar to the localization-delocalization transition in solids.

In this study, we place an artificial lattice defect within the phononic lattice. When the defect is within a phononic bandgap, it hosts spatially localized vibrational modes. We then simulate the pressure dependence of such a defective phononic lattice using two geometries. The first device is similar to the one shown in the manuscript (Supplementary Figure 8a), where we expect the bandgap closing under the application of pressure. The second one is a circular reference device (Supplementary Figure 8b, as studied in previous work). As explained in the main text, the bandgap does not close for this geometry. At zero pressure, the defect mode is localized in both geometries (Supplementary Figure 8). At the same time, we find starkly different behaviour of that mode in circular and rectangular devices under applied pressure (Supplementary Figure 8c,d). In the rectangular device, the mode starts to show displacement over the entire device (highlighted by grey arrows) and thus loses its localization as the bandgap closes. In the circular device, in contrast, the bandgap does not close, and the defect mode always stays within it. Correspondingly, the defect mode remains localized in the entire range of pressures (Supplementary Figure 8d). This further highlights the similarity between phononic and solid-state crystals, despite their very different quasiparticles. We believe that the behaviour we observe can be compared to the localization-delocalization transition.

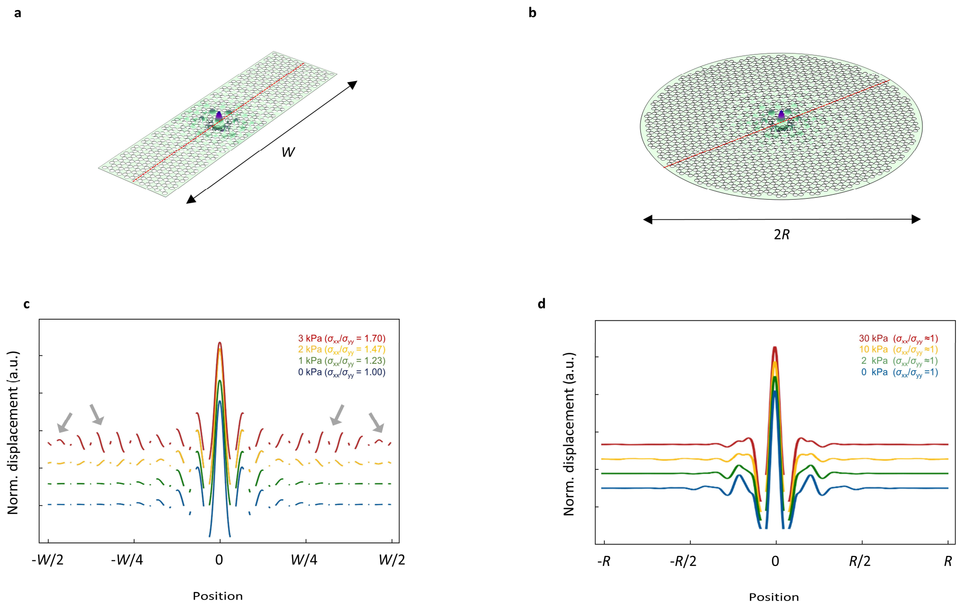

Supplementary Figure 8 | Dephasing a localized defect state using uniaxial tension. a,**b** Mode shape of a localized mode within the bandgap of a rectangular (a) and circular (b) phononic device **c**, Line cut of the normalized displacement extracted along the red line in **a** vs. applied pressure (plots are offset for better visibility). With increased pressure, the degree of uniaxiality is increased and the bandgap gradually closes. At the same time the mode that was localized near device centre at zero pressure becomes delocalized over the entire device. **d,** Same as **c**, but for the circular device shown in (b). Here the mode shape remains virtually unchanged under pressure, as the frequency of the defect mode scales together with the bandgap and maintains its localization.

Supplementary References

1. Kirchhof, J. N. *et al.* Tunable Graphene Phononic Crystal. *Nano Lett.* **21**, 2174–2182 (2021).
